# Generation and Neuronal Differentiation of hiPSCs From Patients With Myotonic Dystrophy Type 2

**DOI:** 10.3389/fphys.2018.00967

**Published:** 2018-07-27

**Authors:** Paola Spitalieri, Rosa V. Talarico, Michela Murdocca, Luana Fontana, Marzia Marcaurelio, Elena Campione, Roberto Massa, Giovanni Meola, Annalucia Serafino, Giuseppe Novelli, Federica Sangiuolo, Annalisa Botta

**Affiliations:** ^1^Medical Genetics Section, Department of Biomedicine and Prevention, University of Rome Tor Vergata, Rome, Italy; ^2^Division of Dermatology, Department of Systems Medicine, University of Rome Tor Vergata, Rome, Italy; ^3^Division of Neurology, Department of Systems Medicine, University of Rome Tor Vergata, Rome, Italy; ^4^Department of Biomedical Science for Health, Policlinico San Donato (IRCCS), University of Milan, Milan, Italy; ^5^Institute of Translational Pharmacology, Italian National Research Council, Rome, Italy; ^6^Istituto Neurologico Mediterraneo (IRCCS), Pozzilli, Italy

**Keywords:** myotonic dystrophy type 2 (DM2), human induced pluripotent stem cells (hiPSCs), intranuclear foci, neuronal population (NP), muscleblind-like 1 and 2 (MBNL1 and 2)

## Abstract

Human induced pluripotent stem cells (hiPSCs)-patient specific are an innovative tool to reproduce a model of disease *in vitro* and summarize the pathological phenotype and the disease etiopathology. Myotonic dystrophy type 2 (DM2) is caused by an unstable (CCTG)n expansion in intron 1 of the *CNBP* gene, leading to a progressive multisystemic disease with muscle, heart and central nervous dysfunctions. The pathogenesis of CNS involvement in DM2 is poorly understood since no cellular or animal models fully recapitulate the molecular and clinical neurodegenerative phenotype of patients. In this study, we generated for the first time, two DM2 and two wild type hiPSC lines from dermal fibroblasts by polycistronic lentiviral vector (hSTEMCCA-loxP) expressing *OCT4*, *SOX2*, *KLF4*, and *cMYC* genes and containing loxP-sites, excisable by Cre recombinase. Specific morphological, molecular and immunocytochemical markers have confirmed the stemness of DM2 and wild type-derived hiPSCs. These cells are able to differentiate into neuronal population (NP) expressing tissue specific markers. hiPSCs-derived NP cells maintain (CCTG)n repeat expansion and intranuclear RNA foci exhibiting sequestration of MBNL1 protein, which are pathognomonic of the disease. DM2 hiPSCs represent an important tool for the study of CNS pathogenesis in patients, opening new perspectives for the development of cell-based therapies in the field of personalized medicine and drug screening.

## Introduction

Myotonic dystrophies (DMs) represent a group of autosomal dominant multisystemic diseases (Harper, 2001), including DM type 1 and type 2 and manifesting highly variability in term of age at onset, severity of the symptoms, and clinical pictures. Myotonic dystrophy type 1 (DM1, OMIM 160900) is one of the most common forms of muscular dystrophy in adults with a prevalence of 1/8000 worldwide (Harper et al., 2002). It is caused by expansion of a CTG trinucleotide repeat in the non-coding region of *myotonic dystrophy protein kinase* gene (*DMPK;* OMIM 605377), a gene located on chromosome 19q13.3 encoding a protein kinase (Brook et al., 1992; Fu et al., 1992; Mahadevan et al., 1992). Myotonic dystrophy type 2 (DM2, OMIM 602688) results from an unstable tetranucleotide CCTG repeat expansion in intron 1 of the *nucleic acid-binding protein* (*CNBP*) gene (previously known as *zinc finger 9* gene, *ZNF9*; OMIM 116955) on chromosome 3q21 (Liquori et al., 2001). The true prevalence of DM2 is still uncertain since DM2 is likely under diagnosed, not only due to the unfamiliarity with the disorder by most clinicians but also to the heterogeneous phenotype and the rather aspecific onset.

The disease mechanism proposed for the pathogenesis of DMs is complex and involves a common RNA gain-of-function mechanism in which the CUG and CCUG repeats cause the accumulation of the expanded transcripts into nuclear RNA foci (Wojciechowska and Krzyzosiak, 2011) which, in turn, sequester and deregulate RNA-binding proteins, such as *MBNL1* and *MBNL2*. These events lead to the impaired alternative splicing (AS) and expression of aberrant embryonic protein isoforms in adult tissues and thus, the spliceopathy observed in DMs is indicated as the main cause of the multisystemic features of the diseases (Ranum and Day, 2004). In addition to spliceopathy, RNA toxicity may trigger further pathogenic mechanisms including dysregulation of miRNAs and RNAi metabolism, RAN translation, defects in protein translation/turnover, activation of cellular stress and apoptotic pathways (Loro et al., 2010; Botta et al., 2013a,b; Meola and Cardani, 2015). To date, defects in the AS of several genes have been reported in DM1 brain, including the glutamate *NMDA* receptor1, *APP*, and *MAPT* genes (Caillet-Boudin et al., 2014). Although, *MBNL2* appears to be a crucial factor in the aberrant splicing of the nervous tissues (Charizanis et al., 2012; Goodwin et al., 2015), little is known about links between the molecular defects and the various CNS symptoms in DM1 patients.

Despite caused by similar mutations, DM1 and DM2 are clinically distinct diseases: they differ for the age at onset, pattern of muscle weakness and absence of congenital and childhood-onset of DM2. Different studies confirm central nervous system (CNS) involvement in DM1 and to a lesser extent in DM2, indicating that DMs can be considered, beyond muscle, true brain disorders (Meola and Sansone, 2007). The cerebral involvement of DM1 patients has been associated with difficulties in executive functions, visuospatial/constructive abilities, memory, facial emotion recognition, and psychomotor delay. In the most severe cases of DM1 (congenital/juvenile form), mental retardation has also been described (Machuca-Tzili et al., 2005; Schara and Schoser, 2006). Furthermore, approximately half of these young patients also have autism spectrum disorders, the frequency of which is related to the number of CTG repeat (Ekstrom et al., 2008). Brain involvement in DM2 is more controversial. There are only few studies regarding the neuropsychological involvement in DM2 pathology and each includes small number of patients (Minnerop et al., 2011). Cognitive manifestations in DM2 include problems with organization, concentration, and word finding which are considerably milder than in DM1 (Romeo et al., 2010). A specific type of “avoidant” personality trait and a significant impairment in executive functions have been observed in both DMs (Meola et al., 2003). Recently, a significant dysexecutive syndrome and impairment of episodic verbal memory have been reported on a large cohort of DM2 patients (Peric et al., 2015), reflecting of frontostriatal and temporal lobe dysfunctions. In contrast to DM1, DM2 has not been associated with developmental abnormalities and thus does not cause severe childhood symptoms. This difference likely explains why no mental retardation similar to that reported in congenital and juvenile forms of DM1 has been described in DM2 patients. The characterization of CNS involvement in DM2 is an evolving field of research, however, no cellular or animal models fully recapitulate the molecular and clinical phenotype of DM2 patients, particularly their brain involvement. The lack of accessibility of neural tissue (both living and post-mortem), combined with difficulties in culturing of these cell types *in vitro*, emphasizes the need for a DM2 cellular system capable to reproduce the mechanisms underlying the CNS impairment in patients.

Human induced pluripotent stem cells (hiPSCs) offer a nearly limitless potential for disease modeling, thanks to their great self-renewal and wide differentiation capacity, coupled with the relative ease of producing patient-specific cells. hiPSCs, carrying mutations implicated in disease, make possible the generation of a wide range of cell types in culture (Yanovsky-Dagan et al., 2015; Spitalieri et al., 2016). Neuronal stem cells (NSCs) derived from Embryonic Stem cells (ESCs) or from hiPSCs have been already generated for DM1, demonstrating their ability to recapitulate important features of the disease, including RNA foci, MBNL sequestration and transcriptional/splicing defects (Marteyn et al., 2011; Denis et al., 2013; Xia and Ashizawa, 2015; Xia et al., 2015; Gourdon and Meola, 2017; Spitalieri et al., 2018).

In this study, we established, for the first time two DM2 and two healthy hiPSC lines as control from human dermal fibroblasts (HDFs), using lentiviral polycistronic vector containing Yamanaka’s four factors (*OCT4*, *SOX2*, *KLF4*, and *c-MYC*). These cells are able to self-renew indefinitely and also to differentiate into neuronal population (NP) maintaining the major specific DM2 hallmarks, such CCTG repeat expansion and CCUG-containing intranuclear RNA foci, which are pathognomonic the disease. The development of a hiPSCs-based platform represents an important tool to study the neurodegenerative and neuromuscular nature of the DM2 mutation and to identify molecular targets for drug design in repeat expansion disorders.

## Materials and Methods

### Skin Biopsy and Culture of Human Dermal Fibroblasts (HDFs)

Two DM2 patients, one male and one female, with a molecular diagnosis of DM2, and two age and sex-matched healthy donors (wild type, WT) have been recruited for dermal biopsy (**Table 1**). Before participation, informed written consent was obtained. The project was approved by The Committees on Health Research Ethics of Tor Vergata Hospital (2932/2017) and in accordance with the Declaration of Helsinki. Skin biopsies (6 mm in diameter) have been digested with 2 mg/mL DISPASE (Sigma-Aldrich, St. Louis, MO, United States) clean off any adipose and epidermal tissue for overnight at +4°C. Tissues have been processed into 0.5 mm cubes, treated with 1 mg/mL COLLAGENASE type I (Sigma-Aldrich, St. Louis, MO, United States) for 4 h at 37°C and successively placed onto 0.1% gelatin-coated (from porcine skin Type A; Sigma-Aldrich, St. Louis, MO, United States) 35-mmculture plates and cultured in primary culture medium DMEM (Sigma-Aldrich, St. Louis, MO, United States) with 15% fetal bovine serum (FBS) (Gibco, Waltham, MA, United States), 1 mM L-Glutamine (Gibco, Waltham, MA, United States), 1% penicillin/streptomycin (Thermo Fisher Scientific, Waltham, MA, United States), 1% non-essential aminoacid solution (Gibco, Waltham, MA, United States), 0.1 mM β-mercaptoethanol (Gibco, Waltham, MA, United States). Fibroblasts growing out from the dermal tissue have been expanded in conventional serum-containing culture media until passage 2.

**Table 1:** Description of samples used in this study.

Samples used in the study							
Donor	Diagnosis	Genotype	Age of biopsy (range)	No of hiPS clonal lines generated	No of hiPS clonal lines used	Passage
C3	Healthy	WT/WT	40–46	21	5	23
C4	Healthy	WT/WT	40–46	17	5	21
D1	DM2	CCTGexp	40–46	13	5	22
D2	DM2	CCTGexp	40–46	7	5	21

### hiPS Cells Reprogramming

A single lentiviral “stem cell cassette” has been used, flanked by loxP-sites (hSTEMCCA-loxP), and encoding for all four reprogramming factors (*OCT4*, *SOX2*, *KLF4*, and *c-MYC*) in a single polycistonic vector (Spitalieri et al., 2015, 2018). Approximately 1.5 × 10^5^ HDFs have been seeded on plastic in 35 mm culture plates and infected in DMEM, containing 15% FBS. The medium has been replaced after 16 h with hiPSCs medium: Dulbecco’s Modified Eagle’s medium-F12 (DMEM/HAM’s F12) (Gibco, Waltham, MA, United States) with 20% knock out serum replacement (KSR) (Gibco, Waltham, MA, United States), 1 mM L-glutamine, 1% penicillin/streptomycin, 1% non-essential amino acid solution, 0.1 mM β-mercaptoethanol and 10 ng/ml of basic Fibroblast Growth Factor (bFGF) (PeproTech, London, United Kingdom) and changed every 2–3 days. hiPSCs colonies have been picked 20–25 days post-infection on the basis of morphology and expanded by plating on mitomycin C-treated MEFs in hiPSCs medium (Sigma-Aldrich, St. Louis, MO, United States). Successively, hiPSCs have been manually picked, passaged on human embryonic stem cell-qualified Matrigel-coated plates (0.05 mg/mL) (Corning, NY, United States) and cultured under feeder-free condition in mTeSR1 medium with Y-27632 ROCK inhibitor (StemCell, Canada), mantaining the stability over 20 and more passages.

### Neural Differentiation of hiPSCs

For neural induction, a modified method previously described was used (Chambers et al., 2009). hiPSCs were cultured in neural induction medium combining dual ALK inhibition (SB + LDN:10 μm SB431542 (Tocris Bioscience, Bristol, United Kingdom) and 0.2 μm LDN193189 (Stemgent, United Kingdom) in KSR media. For differentiating neuronal cells into neurons, increasing amounts of N2 (Gibco, Waltham, MA, United States) (25, 50, and 75%) was added to the KSR media maintaining LDN193189. On day 5–9 sonic hedgehog (SHH; Curis, Lexington, MA United States) was added in N2 media followed by the addition on day 9 of BDNF (PeproTech, London, United Kingdom), ascorbic acid (Sigma-Aldrich, St. Louis, MO, United States) and FGF8 (PeproTech, London, United Kingdom) and maturated on days 12–30 with BDNF, ascorbic acid, GDNF (PeproTech, London, United Kingdom), TGFβ3 (Sigma-Aldrich, St. Louis, MO, United States), cAMP (Sigma-Aldrich, St. Louis, MO, United States) for doparminergic subtype and retinoic acid (Sigma-Aldrich, St. Louis, MO, United States) for motor neuron cells.

### Alkaline Phosphatase (AP) and Immunofluorescence (IF) Analyses

Alkaline phosphatase staining was performed with the Vector Red Substrate Kit (Vector Laboratories, Burlingame, CA, United States^1^) according to the manufacturer’s protocol. For IF analysis, cells were fixed in 4% paraformaldehyde and incubated with the appropriate primary antibodies against OCT4 (Stemgent, United Kingdom), SSEA-4 (Stemgent, United Kingdom), TRA1-60 (Stemgent, United Kingdom), TRA1-81 (Stemgent, United Kingdom), NESTIN (Millipore, Burlington, NJ, United States), PAX6 (Abcam, Cambridge, United Kingdom), OLIG2 (Millipore, Burlington, NJ, United States), CHAT (Millipore, Burlington, NJ, United States), TH (Abcam, Cambridge, United Kingdom), TUJ1 (Covance, Princeton, NJ, United States), MAP2 (Sigma-Aldrich, St. Louis, MO, United States), GFAP (Millipore, Burlington, NJ, United States). Appropriate AlexaFluor 488 or 568 secondary antibodies were incubated for 1 h. Nuclei have been counterstained with 4,6- diamidino-2-phenylindole (DAPI; Sigma-Aldrich, St. Louis, MO, United States). Samples have been observed and acquired by fluorescence microscope (Zeiss Axioplan 2 microscope, Thornwood, NY, United States).

### RNA Fluorescence *in Situ* Hybridization (RNA-FISH) and IF Staining

Intranuclear foci containing (CCUG)_exp_ RNA were dectected in hiPSCs and NPs from DM2 patients using via RNA fluorescence *in situ* hybridization (FISH) using Cy3-labeled (CAGG)_10_ DNA probe (Bonifazi et al., 2006). The cells have been fixed with 4% paraformaldehyde (PFA), permeabilized using 0.3% Triton 100-X, then blocked with 3% BSA and incubated with primary antibodies OCT4, TUJ1, MAP2, GFAP, ISLET1 (Abcam, Cambridge, United Kingdom), LIM3 (Millipore, Burlington, NJ, United States), and MBNL1 (3A4, Santa Cruz Biotechnology, Dallas, TX, United States) labeled with AlexaFluor 488 or 568 secondary antibody. Nuclei have been counterstained with 4,6- diamidino-2-phenylindole. Samples have been observed by confocal microscopy (LEICA TCS SP5). Up to 50 serial sections (each section about 1 μm in thickness), taken from the top to the bottom along the Z axis, have been used to obtain a 3D reconstruction of each analyzed field.

### Gene Expression and Splicing Analysis

RNA from HDFs, hiPSCs and NP was extracted by TRIzol (ThermoFisher Scientific, Waltham, MA, United States) following manufacturer’s instructions and reverse-transcribed with the High-Capacity cDNA Reverse Transcription Kit (Applied Biosystems, Foster City, CA, United States) for cDNA syntesis. Both SYBR Green and TaqMan chemistry with reference genes have been used to quantify mRNA expression. Oligonucleotide sequences and TaqMan probes used are listed in **Table 2**. RT-PCR for *MBNL1* selected target (*FNIP1, MARK2, PPIP5K1, KIF13A, FMNL3, CD47, PALM, ITGA6* genes) was carried out as reported in Venables et al. (2013). Electrophoresis of the PCR products for splicing analysis was carried out on a 2.5% agarose gel.

**Table 2:** List of primers and TaqMan probes used in this study.

Primers	Forward (5′–3′)	Reverse (5′–3′)
5S	TCGTCTGATCTCGGAAGCTAAGCA	AAAGCCTACAGCACCCGGTATT
OCT-4	AACCTGGAGTTTGTGCCAGGGTTT	TGAACTTCACCTTCCCTCCAACCA
SOX-2	AGAAGAGGAGAGAGAAAGAAAGGGAGAGA	GAGAGAGGCAAACTGGAATCAGGATCAAA
NANOG	CCTGAAGACGTGTGAAGATGAG	GCTGATTAGGCTCCAACCATAC
KLF-4	ACGATCGTGGCCCCGGAAAAGGACC	TGATTGTAGTGCTTTCTGGCTGGGCTCC
C-MYC	CGAGAGGACCCGTGGATGCAGAG	TTGAGGGGCATCGTCGCGGGAGGCTG
REX-1	CAGATCCTAAACAGCTCGCAGAAT	GCGTACGCAAATTAAAGTCCAGA
MAP2	CCATTTGCAACAGGAAGACAC	CAGCTCAAATGCTTTGCAACTAT
Olig2	CCTGAGGCTTTTCGGAGC	CTGGCGTCCGAGTCCAT
Peripherin	AGACCATTGAGACCCGGAAT	GGCCTAGGGCAGAGTCAAG
Nestin	CTCTGACCTGTCAGAAGAAT	CCCACTTTCTTCCTCATCTG
GFAP	CCGACAGCAGGTCCATGTG	GTTGCTGGACGCCATTGC
HB9	CACCGAGACCCAGGTGAAGATTT	CCCTTCTGTTTCTCCGCTTCCT
FNIP	GCAGCAGTATTTGTGGGAGTC	TCCAGGCATGTCCATTGG
MARK2	ACCAGCACAAATCGAAGCAG	AGGCAACAGGGACACGCT
PPIP5K1	CCGAATCTTCAGGACTACGC	GGGCATTATGCAGTGTTTCC
KIF13A	TGCCACTTATGGTTGAAGCCA	TGCATCTGACCACCTCTCCCTT
FMNL3	GCGGGAATTTCTGAATGATG	CACTAGGCGGGAGTTCTTCA
CD47	AAGCTGTAGAGGAACCCCTTAATG	GGTCTCATAGGTGACAACCAGTT
PALM	ACAAGCGAGTCTCCAACACG	GTCCGCTTTGTGGATGAGTT
ITGA6	ATCATCCTAGTGGCTATTCTCGC	ACTGTCATCGTACCTAGAGCGT
GAPDH	TTGCCCTCAACGACCACTTTG	CACCCTGTTGCTGTAGCCAAATTC
β-actin	ATTGCCGACAGGATGCAGAA	GCTGATCCACATCTGCTGGAA
CL3N58D	GGCCTTATAACCATGCAAATG	GCCTAGGGGACAAAGTGAGA
*OCT4*-BSP	ATTTGTATTGAGGTTTTGGAGGG	CATCACCTCCACCACCTAAA
β-actin	4326315E	
*MBNL1*	Hs00253287_m1	
*MBNL2*	Hs01058996	
CNBP	Hs00231535_m1	

### Methylation Sensitive High Resolution Melting (MS-HRM) Analysis of the *OCT4* Gene Promoter

Bisulphite treatment of DNA converts all unmethylated cytosines to uracil, leaving methylated cytosines unaltered. Genomic DNA (2 μg in 20 μl) extracted from HDFs and hiPSCs cell lines was treated with EZ DNA Methylation-Gold Kit (Zymo Research, Irvine, CA, United States) according to the manufacturer’s instructions. After bisulfite conversion, the genomic DNA was quantified by the Qubit^®^ 2.0 Fluorometer (Invitrogen, Carlsbad, CA, United States) according to the manufacturer’s instructions. The bisulphite converted DNA was then used for PCR amplification of *OCT4* gene promoter regions with primers designed using Methyl Primer Express Software v1.0 (Applied Biosystem). Primers (OCT4-BSP) were designed to amplify the promoter and exon 1 from −234 to +46 base pairs (NR_034180) (Shi et al., 2013; **Table 2**). PCR amplification and HRM analysis of bisulphite-converted DNA was carried out on 7500 Fast Real-Time PCR System (Applied Biosystems, Foster City, CA, United States), as previously described (Spitalieri et al., 2015).

### Genotyping at the DM2 Locus

Genomic DNA was extracted from DM2 hiPSCs and NPs using Wizard Genomic DNA Purification Kit (Promega, Madison, WI, United States). Molecular characterization of the (CCTG)n expansion in the *CNBP* gene was obtained by a Long-Range PCR based protocol followed by oligospecific hybridization with a (CCTG)_5_ radioactively labeled probe (Botta et al., 2006).

### Statistical Analysis

All values provided for RT-qPCR experiments and for foci analysis are from independent experiments and are reported as mean ± SEM. Each cell line (10 from 2 WT donors and 10 for 2 DM2 donors) has been tested in triplicate and elaborated as mean ± SEM, then for each donor a mean of these value has been further elaborated. Data have been compared using the two-tailed Student’s *t*-test, for independent samples.

## Results

### Generation and Characterization of DM2 and WT hiPSCs

**FIGURE 1:**
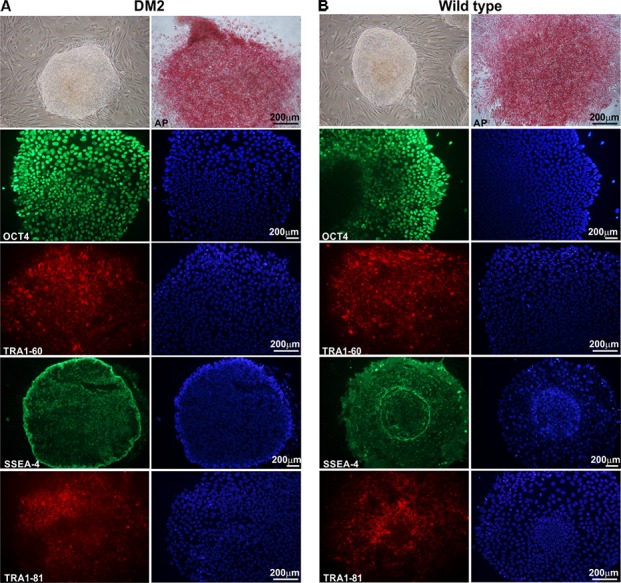
Morphological and immunocytochemical analysis of human hiPSC clones. A representative image, obtained by phase-contrast microscopy, of hiPSCs derived from a DM2 patient **(A)** and from a wild type (WT) **(B)**. Cells have been analyzed at p22 (DM2) and p23 (WT) passages. hiPSCs are cultured under feeder-free condition in mTeSR1 medium, showing undifferentiated morphology, similar to hES cells, with compact refractile and defined borders. hiPSCs express alkaline phosphatase (AP) and are positive for stem cell markers OCT4 (green), TRA1-60 (red), SSEA-4 (green), and TRA1-81 (red). 4,6-Diamidino-2-phenylindole (DAPI) nuclear staining in blue (right column in **A,B**). Scale bars = 200 μm.

**FIGURE 2:**
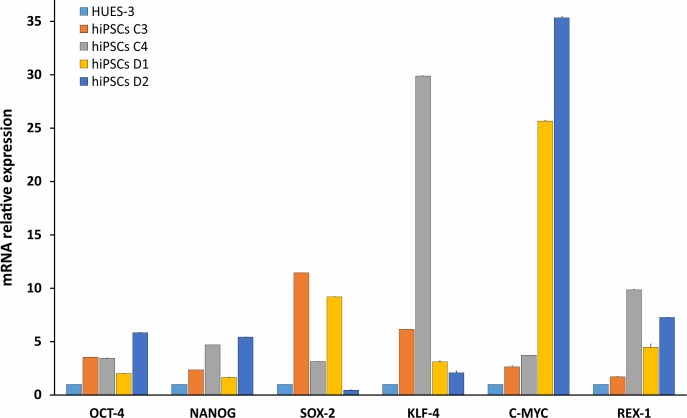
Molecular characterization of stemness markers in hiPSCs. RT-qPCR analysis of *OCT4*, *NANOG*, *SOX-2*, *KLF-4*, *c-MYC*, and *REX-1* transcripts evaluated in WT (C3 and C4) and DM2 (D1 and D2) hiPSCs. The values have been normalized respect to those obtained in a human embryonic stem cell line (HUES-3). 5S ribosomal gene is used as reference. Data are from independent experiments (*n* = 5 clones for each donor indicated) and represented as mean ± SEM.

**FIGURE 3:**
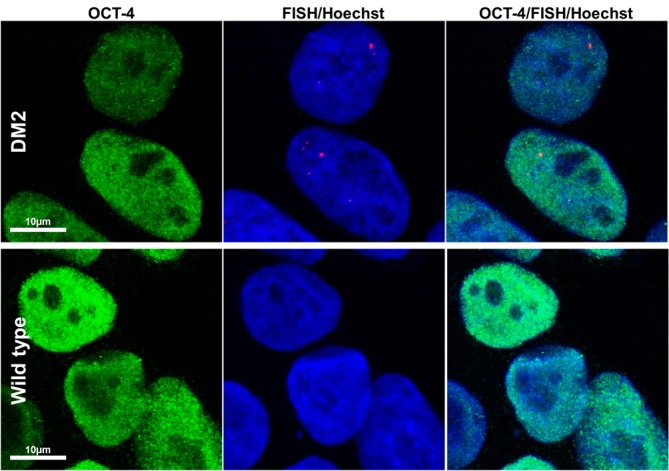
RNA-FISH in hiPSCs. Representative confocal microscopic images showing the presence of CCUG-containing RNA foci (red) in DM2 nuclei, positive for stemness marker OCT-4 (green). Cells have been analyzed at p22 (DM2) and p21 (wild type) passages. Foci have been detected in 100% of DM2 hiPSCs, whereas no foci have been detected in WT hiPSCs. 4,6-Diamidino-2-phenylindole (DAPI) nuclear staining (blue). Scale bars = 10 μm.

HDFs from two DM2 patients and two healthy controls (WT) were reprogrammed using a single lentiviral “stem cell cassette” (hSTEMCCA-loxP), containing all defined genes necessary to obtain hiPSCs (Spitalieri et al., 2015, 2018). Thirty-eight hiPSC clonal lines from two healthy donors (WT) and 20 hiPSC clonal lines from two DM2 patients have been generated and 5 lines/each donor have been used (**Table 1**), molecular and IF analyses have been performed between passages p20 and p23 in culture. All hiPSCs exhibited an undifferentiated morphology with compact refractile, defined borders and high rate of proliferation. As shown in **Figure 1**, cells expressed alkaline phosphatase activity (AP), the transcription factor OCT4 and surface stemness markers such as TRA 1–60, SSEA-4, TRA 1–81. The success of reprogramming has been also confirmed by RT-qPCR analysis, carried out using primers designed to specifically detect endogenous reprogramming transcription factors (*OCT4, NANOG, SOX2, KLF4, c-MYC, REX-1*) and to elude any possible contamination of exogenous transcripts expressed by the hSTEMCCA-loxP lentiviral vector. The results revealed a clear expression of embryonic marker genes in both DM2 and WT hiPSCs, compared to those detected in human embryonic stem cells (HUES-3) (**Figure 2**). No chromosomal aberrations in DM2 and WT hiPSCs have been observed by karyotype analysis (data not shown). Moreover, RNA-FISH, combined with IF analysis, revealed the presence of intranuclear CCUG-containing foci in OCT4-positive hiPSCs derived from DM2 patients, thus confirming the retention of expanded sequences accumulated within nuclei of pluripotent cells. No foci were detected in the healthy controls-derived hiPSCs (**Figure 3**).

### Methylation Analysis of the *OCT-4* Promoter

**FIGURE 4:**
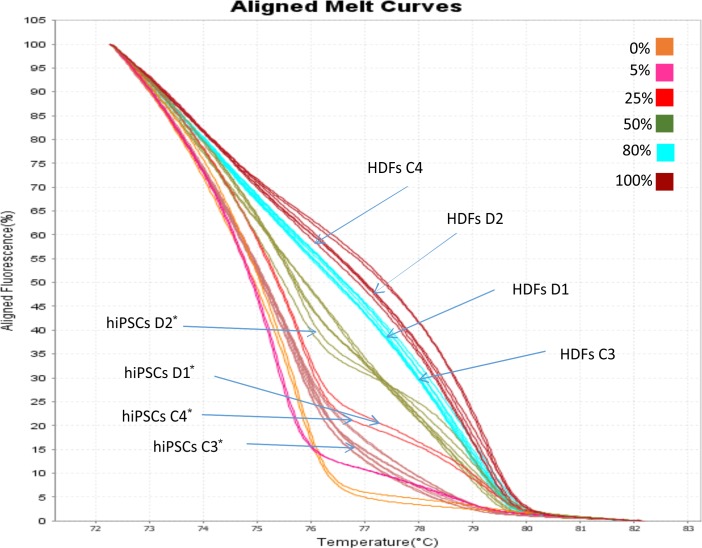
MS-HRM analysis of the *OCT4* promoter region. Standard aligned melting curve corresponding to 0, 5, 25, 50, 80, and 100% of methylation. Aligned melting curves of hiPSCs and HDFs derived from DM2 patients (D1 and D2) and healthy control (C3 and C4). Student’s *t*-test was used for statistical analysis. ^∗^*P* < 0.005.

Methylation Sensitive High Resolution Melting (MS-HRM) analysis has been used to examine if the *OCT4* expression observed in hiPSCs lines was correlated with the hypomethylation of minimal promoter and exon 1 of the gene. Eleven CpG dinucleotides have been analyzed within this region. The aligned melt curve profiles of standard with different ratios of methylated-to-unmethylated templates (0, 10, 25, 50, 80, and 100%), HDFs and hiPSCs lines are displayed in **Figure 4**. The methylation pattern of the *OCT4* region analyzed differs significantly between HDFs and hiPSCs lines. The percentage of *OCT4* methylation in both WT and DM2 HDFs (values ranging from 70 to 90%) resulted to be significantly higher respect to WT and DM2 hiPSCs (values ranging from 20 to 40%; *p* < 0.005) (**Figure 4**). These data are in agreement with the activation of the *OCT4* gene that occurs along the HDFs to hiPSCs reprogramming process.

### *MBNL1* and *MBNL2* Are Involved in Pluripotent Stem Cell Reprogramming of WT and DM2 Lines

Previous studies have shown that both *MBNL1* and *MBNL2* have a lowest relative mRNA level in ESCs/hiPSCs compared to other cells and tissues (Han et al., 2013; Venables et al., 2013), confirming a conserved and prominent role for both genes in ESC-differential AS. In fact, MBNL proteins are shown to negatively regulate an ESC-differential AS network that controls pluripotency and reprogramming (Han et al., 2013). We therefore performed a gene expression analysis by RT-qPCR in order to quantify mRNA levels of *MBNL1* and *MBNL2* genes in our WT and DM2 HDF and hiPSC lines. Results clearly showed a variation in the expression of these genes among the four analyzed cell lines, independently from their genotypes.

**FIGURE 5:**
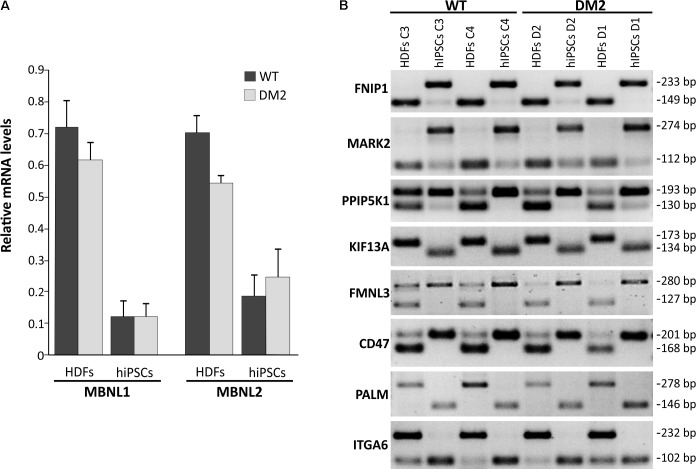
RT-qPCR analysis of *MBNL1* and *MBNL2* gene and alternative splicing assay. **(A)** RT-qPCR assay for *MBNL1* and *MBNL2* genes expression in the HDFs to hiPSCs transition of WT and DM2 lines. Gene expression levels reported are an average of the WT and DM2 cell lines normalized to β-actin used as reference gene. **(B)** Eight-switch-like splicing changes of genes regulated by MBNL1 protein have been assessed by conventional RT-PCR analysis during the HDFs to hiPSCs transitions in WT and DM2 lines. The upper and lower bands on each gel represent the long (exon-included) and short (exon-omitted) isoforms, respectively.

In particular, RT-qPCR assay revealed that HDFs express the higher levels of *MBNL1* and *MBNL2* transcripts (average values of DM2 and WT cell lines: 0.648 ± 0.07 and 0.603 ± 0.03, respectively) compared to hiPSCs (average values of DM2 and WT cell lines: 0.115 ± 0.04 and 0.206 ± 0.07, respectively) (**Figure 5A**). These data confirm that *MBNL2*, as well as *MBNL1*, is differently regulated during hiPSCs reprogramming and plays a central and negative regulatory role in pluripotency. However, comparison of the *MBNL1* and *MBNL2* expression pattern in WT (C3 and C4) and DM2 (D1 and D2) HDFs and hiPSCs did not revealed any significant differences.

We then focused our attention on a set of genes (*FNIP1*, *MARK2*, *PPIP5K1*, *KIF13A*, *FMNL3*, *CD46*, *PALM*, and *ITGA6*), whose AS is controlled by the MBNL proteins during the HDFs to hiPSCs transition (Venables et al., 2013). As expected, a classical HDFs to hiPSCs switch-like splicing pattern has been observed for all the gene analyzed in both DM2 and WT cell lines (**Figure 5B**). Again, no significant differences have been observed among DM2 and WT cells, thus implying that the MBNLs sequestration into CCUG-containing ribonuclear foci does not perturb splicing changes upon the induction of the original HDFs into the pluripotent state.

### Differentiation and Characterization of hiPSCs-Derived Neural Population (NPs)

**FIGURE 6:**
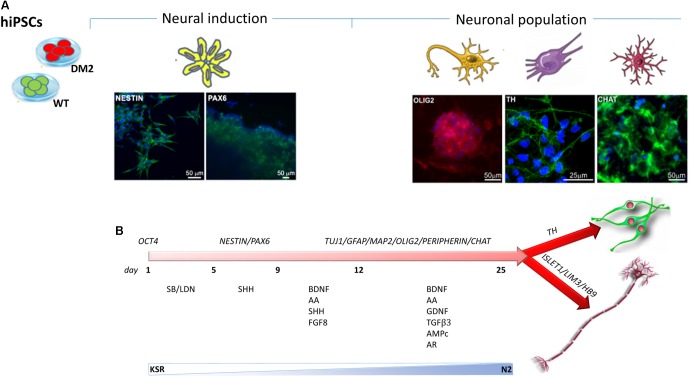
Timeline of hiPSCs differentiation into NPs. **(A)** Immunostaining has been performed using antibodies specific to neural stem cell markers NESTIN (green) and PAX6 (green) and to specific markers for NP as OLIG2 (red), CHAT (green), and TH (green). 4,6-Diamidino-2-phenylindole (DAPI) nuclear staining in blue. Scale bars = 50 and 25 μm. **(B)** Neuronal differentiation is obtained by SMAD signaling modulation via treatment with SB431542/LDN. The timing of differentiation represents the day at which several factors have been added and the crucial stages of differentiation from hiPSCs to NPs through the neural induction.

Neuronal differentiation was performed using EB-based approach by DUAL inhibition of SMAD signaling, obtained with the addition of SB431542/LDN to achieve full neural conversion from WT and DM2 hiPSCs into NPs. The expression of neural stem cell marker, NESTIN, and dorsal forebrain marker, PAX6, showed a profile of neural induction after the first 12 days of the differentiation process, as revealed by immunofluorescence analysis (**Figure 6A**). Following the addition of several factors such as BDNF, ascorbic acid, TGFβ3, GDFN, retinoic acid, and cAMP to neural precursors, at day 25 of differentiation, we observed a mixed population of cells expressing different neuronal markers specific for neurons, astrocytes, oligodendrocytes, early dopaminergic cells and early motor neuron precursors. After day 25, we obtained neuronal networks formed by precursors of oligodendrocytes and dopaminergic cells, as demonstrated by positive staining of OLIG2 and Tyrosine Hydroxylase (TH) antigen, respectively (**Figure 6A**). Cells also showed positivity for choline acetyltransferase marker (CHAT), one of the enzymes involved in the synthesis of the neurotransmitter acetylcholine (**Figure 6A**). The differentiation process has verified according to the protocol described in **Figure 6B**.

**FIGURE 7:**
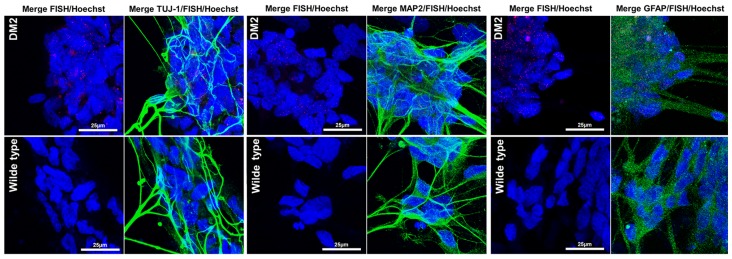
RNA-FISH in NPs. Representative confocal image of the analysis for intranuclear foci (red) and neuron marker β-tubulin 3 (TUJ1, green), dendritic marker (MAP2, green), and astrocytes marker GFAP (green) performed on DM2 and WT NPs. 4,6-Diamidino-2-phenylindole (DAPI) nuclear staining (blue). Image show the presence of CCTG RNA foci only in DM2 nuclei. Scale bars = 25 μm.

**FIGURE 8:**
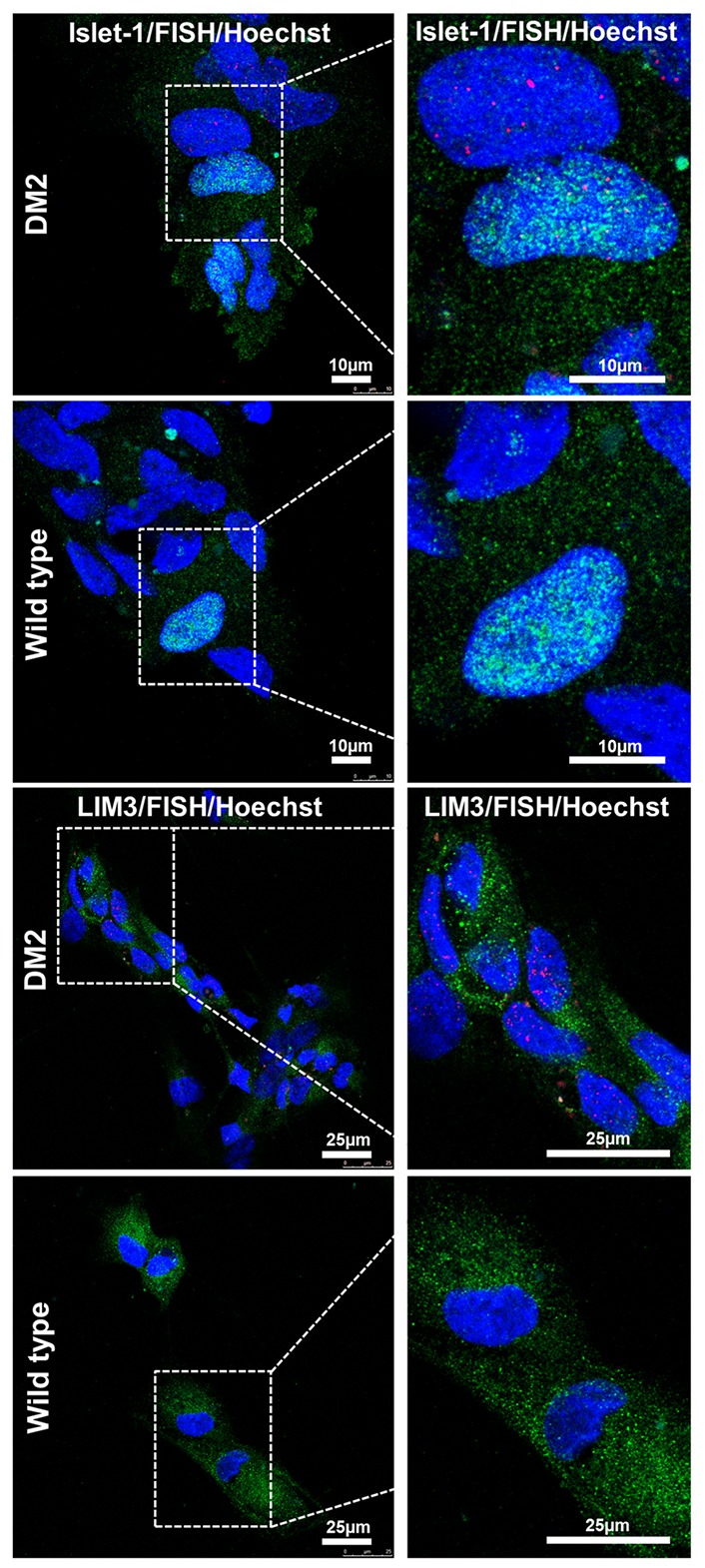
RNA-FISH in precursor motoneurons. Representative confocal image of immunolabeling showing positive signaling for ISLET (green) and LIM3 (green), representing motor neuron precursor markers and intranuclear CCTG repeat foci (red) in DM2 cells. No foci have been detected in WT ones. 4,6-Diamidino-2-phenylindole (DAPI) nuclear staining (blue). Scale bars = 25 μm.

**FIGURE 9:**
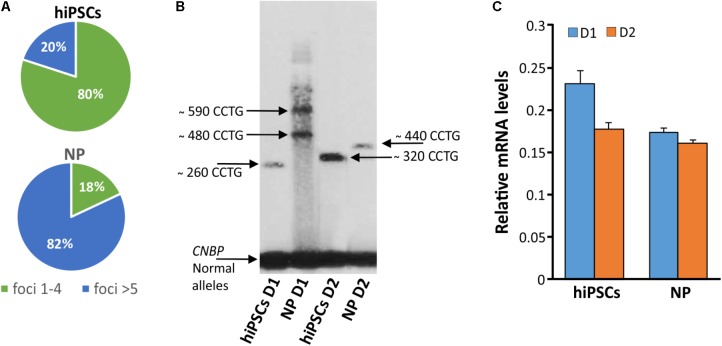
Detection of DM2 mutation at DNA, expression of *CNBP* gene and RNA level during differentiation. **(A)** Percentages of nuclear foci (1–4 or >5) in hiPSCs and NP showing the increase of foci number along their differentiation process. **(B)** LR-PCR followed by hybridization with a (CTG)_5_-radioactively labeled probe on DNA extracted from DM2 hiPSCs and NPs, arrows indicated *CNBP* normal and expanded alleles. **(C)** RT-qPCR assay for *CNBP* expression in DM2 hiPSCs and NPs. β-actin is used as reference gene.

In order to test if NPs from DM2 patients retain the main molecular hallmark of the disease after the differentiation process, we evaluate the presence of intranuclear CCUG-containing RNA foci in cells expressing known neuronal markers. RNA-FISH, combined with IF analysis in DM2 and WT NPs, showed ribonuclear foci only in DM2 cells, positive to neuron-specific marker TUJ1, dendritic marker MAP2 and astrocytic marker GFAP (**Figure 7**). A further differentiation was obtained by adding retinoic acid until the appearance of motor neuron cells, in which ISLET1 and LIM3 markers together with RNA-foci have been detected by RNA-FISH. No foci were present in WT motor neurons (**Figure 8**). The quantification of ribonuclear foci in DM2 hiPSCs and NPs showed that the number of foci appears to be directly related with the neuronal differentiation. As shown in **Figure 9A**, the percentage of cells containing a number of foci/nucleus >5 was significantly higher in DM2 NPs respect to DM2 hiPSCs (82 and 20% of hiPSCs, respectively). We then wanted to verify if the observed increase in the number of CCUG-containing RNA foci could be related to the DM2 mutation size and/or *CNBP* expression. LR-PCR analysis on DNA extracted from DM2 hiPSCs and NPs revealed that in neural cells the number of CCTG repetition increases compared to hiPSCs in both lines (**Figure 9B**). Conversely, RT-qPCR showed variable expression levels of *CNBP* transcript with no significative differences between hiPSCs (median value normalized to β-actin gene: 0.203 ± 0.01) and NP DM2 cell lines (median value normalized to β-actin gene: 0.166 ± 0.04) (**Figure 9C**).

**FIGURE 10:**
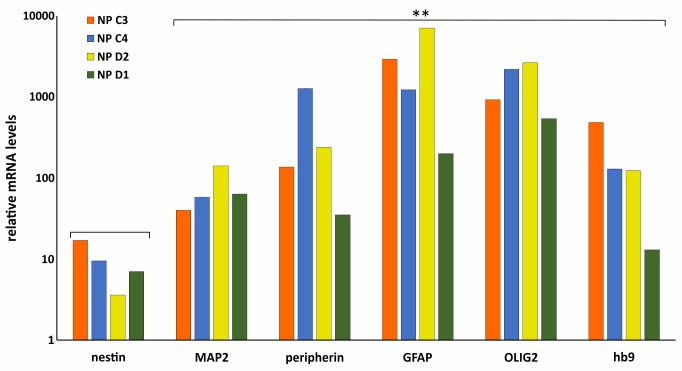
Molecular characterization of NP. RT-qPCR analysis of *Nestin*, *MAP2*, *peripherin*, *GFAP*, *OLIG2*, and *HB9* in WT (C3 and C4) and DM2 (D1 and D2) NPs. Data are from independent experiments (*n* = 5 clones for each donor indicated) and represented as mean ± SEM; (^∗∗^*p* < 0.01). *GAPDH* is used as reference gene. Statistical analysis refers to *Nestin* gene expression.

**FIGURE 11:**
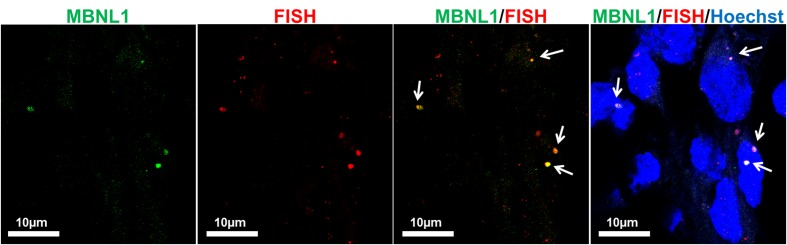
Co-localization of CCUG-containing ribonuclear foci with MBNL1 protein. Representative confocal microscopic images showing the presence of RNA-FISH of foci (red) co-stained with the splicing factor MBNL1 (green) in DM2- derived neuron (D2). 4,6-Diamidino-2-phenylindole (DAPI) nuclear staining (blue). Scale bars = 10 μm.

To further validate the differentiation capacity of DM2 and WT hiPSCs into NPs, we measured the expression levels of specific neural markers by RT-qPCR. This analysis revealed that NESTIN, an early marker of neural stem cell, resulted to be less expressed respect to markers of mature neurons, such as astrocytes and oligodendrocytes in both DM2 and WT cells. Specifically, after day 25th induction of neuronal and astrocytic differentiation resulted in transcriptional boost of lineage specific markers (i.e., MAP2, GFAP, peripherin). At the same time point of the *in vitro* differentiation, we also observed an evident expression level of both oligodendrocyte transcription factor OLIG2, playing a crucial role in the neurogenesis, and of HB9, an early motor neuron marker (*p* < 0.01) (**Figure 10**). These data demonstrated a robust patterning response in SB431542/LDN-treated neural progeny and derivation of relevant neuron subtypes after short differentiation periods (∼25–30 days) compared to 40–50 days necessary for inducing rapid and complete neural conversion, as already reported (Perrier et al., 2004; Lee et al., 2007). Finally, the co-localization of ribonuclear inclusion with MBNL1 protein typical of DM2 mucle cells (Cardani et al., 2014) is also present in DM2 NPs (**Figure 11**), showing that our neuronal cells are a functional DM2 modeling tool.

## Discussion

The majority of study in DM2 have been focused on the muscle pathology, but less is known about the impact of the CCTG expansion on the rest of the body. CNS symptoms, such as intellectual disability, behavioral issues and day time sleepness also have a large impact on patient’s quality of life. However, the CNS is especially difficult to study as most material can only be obtained post-mortem. In this study we report, for the first time, that neural cells can be obtained by DM2 patient-specific hiPSCs, opening new avenues for modeling this complex multisystemic disease.

We obtained two hiPSCs from a male and a female affected by DM2. The analyses have been performed by comparing them to WT hiPSCs age- and sex-matched (**Table 1**). No isogenic lines were produced, but in order to improve the statistical significance, 5 cell lines from each patients, for a total of 10 lines DM2 and 10 lines WT, were always compared, considering cell-to-cell variability. In this way, even if partially, we have circumvented the limitation due to the lack of isogenic cells, in which a gene targeting approach has reversed the pathological phenotype (Spitalieri et al., 2018). The differentiation protocol used in the present study has considered the treatment with small molecules, SB and LDN that, effectively enhanced the differentiation of hiPSCs and changed their state toward a transitional EB state, inducing *in vitro* neural maturation. As previously described, the inhibition of BMP and TGF-β signaling by the small molecules early during hiPSCs differentiation selectively blocks endodermal and mesodermal cell fates. This induces default neural specification, termed dual-SMAD inhibition, dramatically enriches neural ectodermal directly from pluripotent cells (Chambers et al., 2009; Sances et al., 2016). The following steps correspond to the neutralization through dual-SMAD inhibition, caudalization and ventralization through SHH activation by SHH protein and the consequent neural determination by neutrophic factors such as BDNF, GDNF, TGFβ3, and others for promoting neural maturation and survival. The robustness and modularity of the above strategy beyond hiPSCs differentiation offered an efficient defined and valid platform for the rapid generation of neural cell types including somatic motor neurons and dopamine neurons, as demonstrated by expression analyses of neural markers. Moreover, after differentiation no difference in term of differentiation efficiency has been observed among DM2 and WT cells. NPs obtained in this study typically contain heterogeneous cell progeny containing original neural stem cells (NSCs), including NSCs themselves and their progeny (Hawes and Pera, 2006), showing a similar response to induction factors during time-course.

The main pathognomonic molecular hallmark of DM2, represented by the presence intranuclear accumulation of CCUG-containing foci which sequester MBNL1 protein, are also conserved after neuronal differentiation. This suggests that RNA toxicity underlay CNS dysfunction in DM2 patients and allowed us to consider our hiPSCs-derived NP cells as a good disease model. Interestingly, in DM patients the most severely affected tissues are those mainly formed by non-cycling cell populations (i.e., myotubes, neurons, and cardiomyocytes), on the contrary, self-renewing tissues are much less affected (Giagnacovo et al., 2012). This observation indicates that in these cells foci formation and the sequestration factors essential for RNA splicing and processing would be a continuous and progressive phenomenon, eventually leading to the onset of disease symptoms. The quantification of ribonuclear foci during the neural differentiation of our DM2 hiPSCs showed that the number of foci increased after the neuronal differentiation and is not related to the number of passages in culture which is maintained after hiPSCs to NP transition. This observation is in accordance with previous data indicating that the number and density of ribonuclear foci progressively increase in cells that stop dividing and undergo differentiation (Cardani et al., 2009). Nevertheless, the expression level of the *CNBP* transcript does not differ significantly between hiPSCs and NP from DM2 patients. It is therefore possible that in non-cycling NP cells ribonuclear foci do not undergo relocation and degradation occurring during mitosis and progressively increase in number and size, due to a continuous accumulation of both expanded RNAs and protein factors.

Another important aspect still unknown in DM2 is the dynamic of the (CCTG)n expansion in different cell types and the mechanism causing the (CCTG)n expansion and/or contraction. The CCTG repeats expansion is highly unstable and tends to increase with age, while, differently from DM1, it usually decreases on transmission to the next generation being shorter in the children (Meola and Cardani, 2015). The cause for the instability is probably related to an *Alu*-mediated mechanism developed into the large CCTG expanded tract or to unequal crossing over (Dere et al., 2004; Kurosaki et al., 2012). This instability leads to the somatic mosaicism that gives rise to intra-tissue, inter-tissue, and cell-type variability over a patient’s lifetime. Differently from DM1, the CCTG repetition number is not associated with phenotype severity, age, onset of disease. All together, these observations could explain some distinct features of DM2 such as the lack of a congenital form and anticipation and the later onset of symptoms (Udd et al., 2003). Interestingly the determination of the CCTG repetition number in our DM2 cells lines showed an increased in the expansion size during the neural differentiation. Of course, we are aware of all the limitations in determining the DM2 mutation size by LR-PCR, however, this analysis indicates a trend toward the expansion in our neuronal cell model which could be further investigated at molecular level.

Consistent with previous studies, we found that the *MBNL1* and *MBNL2* genes are expressed at minimal levels in hiPSCs compared to fibroblasts, confirming that both MBNL proteins negatively regulate pluripotency and reprogramming (Han et al., 2013). The direct consequences of MBNL downregulation in hiPSCs is the switch-like splicing pattern of a set of genes whose AS is controlled by the MBNL proteins during the HDFs to hiPSCs transition (Venables et al., 2013). Interestingly, no differences have been observed in the splicing profile these MBNL-dependent genes in WT and DM2 cell lines. Consistently, once established, either DM2 and WT hiPSCs grew in a similar pattern with a high nuclear to cytoplasm ratio, expressed stem cells markers and had stem cells features of self-renewal and pluripotency. Taken together, our results indicate that sequestration of the MBNL proteins into the ribonuclear foci of DM2 cells does not exert a toxic effect on the reprogramming and differentiation capacity into NPs of hiPSCs.

Multiple RNA-based approaches have been investigated to induce the downregulation of the *DMPK* expanded transcripts (Thornton et al., 2017) and recently CRISPR/CAS9 genome editing technology has been successful applied as a gene therapy approach for DM1 (Provenzano et al., 2017). The DM2-hiPSCs generated in our work could represent a powerful tool to study the potential role of genome editing and RNA-based therapeutic approaches also for DM2 pathology. Moreover, the detection of specific markers expressed in DM2 NPs could allow to follow the time-course of neurogenesis gaining further insights into the development of brain impairment in patients. The understanding/elucidation of the functional alteration affecting DM2 neural cells would help the finding of the more suitable pharmacological intervention to correct such abnormalities and would thus lay the bases for a future patient-tailored therapy.

## Author Contributions

AB, FS, PS, and GN conceived the experiments. PS, RT, MiM and MaM conducted the experiments. EC, GM, and RM selected patients, LF and AS analyzed the data. AB, FS, and PS wrote the manuscript. All authors discussed and reviewed the manuscript.

## Conflict of Interest Statement

The authors declare that the research was conducted in the absence of any commercial or financial relationships that could be construed as a potential conflict of interest. The handling editor declared a shared affiliation, though no other collaboration, with several of the authors at time of review.

## Footnotes

1 www.vectorlabs.com
